# Postcholecystectomy Duodenal Injury: Role of Conservative Management

**DOI:** 10.7759/cureus.11144

**Published:** 2020-10-24

**Authors:** Subhash Soni, Ashish Swami, Taruna Yadav, Kelu S Sreesanth, Vaibhav K Varshney

**Affiliations:** 1 Surgical Gastroenterology, All India Institute of Medical Sciences, Jodhpur, Jodhpur, IND; 2 Radiology, All India Institute of Medical Sciences, Jodhpur, Jodhpur, IND

**Keywords:** cholecystectomy, duodenal injury, conservative

## Abstract

Postcholecystectomy duodenal injuries are very rare complications. Early surgical intervention is a common practice due to its fatal consequences. Most of the patients with post laparoscopic cholecystectomy duodenal injury reported in literature have been successfully managed by early surgical repair. We present here a case of a 32-year-old female who underwent open cholecystectomy and had an injury in the second part of the duodenum. She was subsequently managed conservatively.

## Introduction

Postcholecystectomy duodenal injuries are uncommon but can have fatal complications. Early detection and surgical management are required for a favorable outcome. However, few cases of post laparoscopic cholecystectomy (LC) duodenal injury have been reported with successful conservative management. We present a case report of the successful conservative management of post open cholecystectomy duodenal injury. To our knowledge, this is the first case report of post open cholecystectomy duodenal injury, and our experience is reported herein.

## Case presentation

A 32-year-old woman presented with concerns of intermittent right upper abdominal pain for one year. Her liver function tests were within normal limits. Ultrasonography (USG) revealed cholelithiasis along with choledocholithiasis. Endoscopic retrograde cholangiopancreatography (ERCP) revealed two small filling defects, and we extracted the stone via stent placement in the common bile duct (CBD). She underwent open cholecystectomy one month following ERCP. Intraoperatively, we noted the gall bladder (GB) was contracted and filled with multiple calculi, the CBD was dilated, and the hepatoduodenal ligament was frozen. Intraoperatively there was no suspicion of any bowel injury. An abdominal drain was placed in the right subhepatic region. She was discharged on postoperative day (POD) three with a drain in situ due to persistent serosanguinous discharge. The abdominal drain was removed on POD eight as output was decreased. A GB biopsy showed features of chronic cholecystitis.

Following drain removal the next day on POD nine, she had concerns of abdominal pain and bilious vomiting. She was admitted and started on conservative management with nasogastric (NG) tube placement. USG of the abdomen revealed an 8 cm x 3 cm fluid collection in the GB fossa with multiple localized thick collections on the right side of the abdominal cavity. Given the sub-hepatic collection and localized peritonitis, we inserted an abdominal drain into the collection with the patient under local anesthesia. We drained 1.4 L of biliopurulent discharge on day one. She improved symptomatically but had persistent 500 to 600 mL bilious discharge from the drain and high NG output, so continued to be managed conservatively.

**Figure 1:**
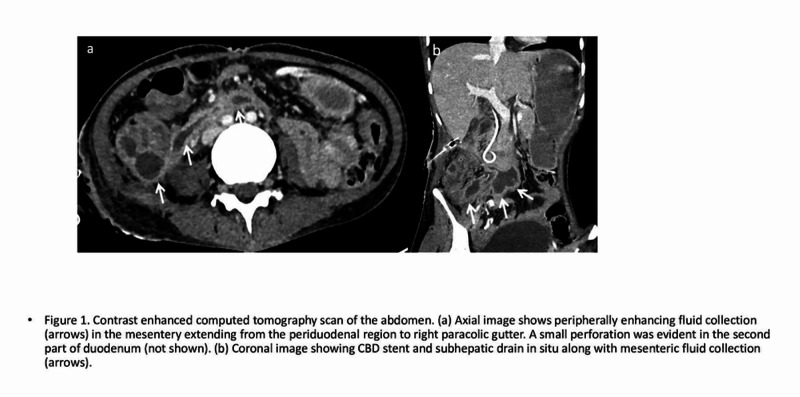
CECT Abdomen CECT - Contrast-enhanced computed tomography

Given the suspected CBD/duodenal injury, she was referred to our center on POD 23 for further management. On initial examination, the patient’s vitals were stable with NG output of 500 to 600 mL/day, and the abdominal drain was draining 400 to 500 mL of bile each day. Blood investigations showed leucocytosis with normal liver function tests. Contrast-enhanced computed tomography (CECT) of the abdomen revealed multiloculated collection in the para duodenal region extending into the right paracolic gutter with a small perforation in the second part of the duodenum with abdominal drain and CBD stent in situ (Figures 1A, 1B). She was given a trial of oral charcoal, which confirmed the presence of duodenal injury. Because of her delayed clinical presentation along with CECT findings, we planned for conservative management. Her drain was gradually withdrawn, and its output gradually decreased to 30 to 40 mL/day over one week. The abdominal drain was removed 30 days following insertion. Subsequently, her NG output also decreased, following which she was allowed oral liquids, which she tolerated well. She was discharged after a total hospital stay of one month, and the patient now has a normal follow-up course after six months.

## Discussion

LC is now the gold standard procedure for symptomatic gallstones disease. However, conversion to open cholecystectomy may be needed in 5% to 10% of cases [1,2]. Bowel injuries are rare, fatal complications of cholecystectomy. Duodenum injury is the most common form of bowel injury during cholecystectomy, reported in 0.05% to 0.14% of cases with a mortality rate ranging from 8.3% to 75% [3,4].

Thermal injuries are attributed as the major mechanism for duodenal injury during cholecystectomy. However, traction injury and drains causing duodenal perforations have also been reported in the literature [4,5]. The reason for the duodenal injury in our patient was not known as she was a referred case, and notes from the operation were not helpful. All these mechanisms could not be completely ruled out as transmural thermal duodenal injury may present up to 16 days postoperatively. Also, this patient was discharged with a drain in situ. The most common site of injury is the second part of the duodenum (60%), followed by the first part (30%), and the third part (5% to 10%) [4].

The timing of missed bowel injury presentation may vary from day zero up to two to three weeks. The literature suggested that most of the duodenal injuries were diagnosed and repaired intraoperatively. Machado et al. showed that 46% of duodenal injuries were detected immediately on the table [4]. The mean period of detection of bowel injury reported in the literature varies from 1.7 days to three days postoperatively [4,5]. A few cases with conservative management for duodenal perforation also have been reported in the literature (Table 1). Modi et al. described a case report of post LC duodenal injury diagnosed on POD two and managed via ERCP-guided CBD stenting and drainage of the duodenal leak [6]. Jing et al. reported post LC duodenal leak on POD four that was managed conservatively [7]. Notably, both patients had no sign of peritonitis or sepsis. Also, Gaillard et al. reported endoscopic duodenal stent placement with pigtail drainage for collection following post LC duodenal perforation [8].

**Table 1:** Case reports with postcholecystectomy duodenal injury and conservative management ERCP - Endoscopic retrograde cholangiopancreatography; CBD - Common Bile Duct

S. No.	Author	Age/Sex	Indication of surgery	Technique of surgery	Time interval surgery and diagnosis of duodenal injury	Management	Length of hospital stay
1.	Modi et al [6]	47 years/ Male	Chronic cholecystitis status post ERCP CBD stone removal	Laparoscopic	2 days	ERCP guided CBD stenting and drainage of duodenal leak	21 days
2.	Jing et al [7]	74 years/ Male	Gall stone disease	Laparoscopic	4 days	Conservative	26 days
3.	Gaillard M et al [8]	66 years/ NA	Acute cholecystitis	Laparoscopic	Intraoperative	endoscopic duodenal stent placement with pigtail drainage for collection	NA
4.	Present case	32 years/ Female	Gall stone disease status post ERCP CBD removal	Open	9 days	Drain placement	30 days

The literature suggests that late presentation of postcholecystectomy duodenal injury is associated with poor outcomes, mainly due to the presence of sepsis and peritonitis. Machado et al. reported that intraoperative detection of duodenal injury was associated with 100% survival, whereas detection on POD one, two, and three or more was associated with 66%, 50%, and 66% survival, respectively [4]. Our patient also had delayed presentation for duodenal injury around eight days after surgery, but the presence of localized peritonitis and achievement of controlled duodenal fistula via drain placement produced a successful outcome in the conservative management of duodenal injury.

However, the high mortality rates due to duodenal injuries are still a concern [3,9,10]. Huang et al. and Deziel et al. reported 21% and 8.3% mortality rates, respectively, in post LC duodenal injuries [3,9]. As stated earlier, immediate detection of duodenal injury and primary repair of the injury site is associated with a good prognosis; every attempt must be made for early detection of bowel injury.

## Conclusions

Duodenal injuries are a rare but fatal complication of cholecystectomy. Most of the injuries are diagnosed and managed intraoperatively but delayed presentation has been also reported. Thermal and traction injuries are attributed as the major mechanism for duodenal injury during cholecystectomy. Early detection of bowel injury and surgical intervention is associated with a good outcome. Conservative management with controlled drainage of the duodenal leak may be tried in patients with localized peritonitis. A multidisciplinary approach and management at an experienced high volume center is required for a better outcome.

## References

[1] Livingston EH, Rege RV (2004). A nationwide study of conversion from laparoscopic to open cholecystectomy. Am J Surg.

[2] Keus F, Gooszen HG, van Laarhoven CJ (2010). Open, small-incision, or laparoscopic cholecystectomy for patients with symptomatic cholecystolithiasis. An overview of cochrane hepato-biliary group reviews. Cochrane Database Syst Rev.

[3] Huang X, Feng Y, Huang Z (1997). Complications of laparoscopic cholecystectomy in China: an analysis of 39,238 cases. Chin Med J (Engl).

[4] Machado NO (2016). Duodenal injury post laparoscopic cholecystectomy: Incidence, mechanism, management and outcome. World J Gastrointest Surg.

[5] Testini M, Piccinni G, Lissidini G (2008). Management of descending duodenal injuries secondary to laparoscopic cholecystectomy. Dig Surg.

[6] Modi M, Deolekar S, Gvalani A (2014). An option of conservative management of a duodenal injury following laparoscopic cholecystectomy. Case Rep Surg.

[7] Jing K, Shuo-Dong W (2014). Postoperative delayed duodenum perforation following elective laparoscopic cholecystectomy. Case Rep Med.

[8] Gaillard M, Dupond-Athenor A, Donatelli G, Dagher I (2017). Conservative endoscopic management of a large duodenal defect after cholecystectomy. J Visc Surg.

[9] Deziel DJ, Millikan KW, Economou SG, Doolas A, Ko ST, Airan MC (1993). Complications of laparoscopic cholecystectomy: a national survey of 4,292 hospitals and an analysis of 77,604 cases. Am J Surg.

[10] El-Banna M, Abdel-Atty M, El-Meteini M, Aly S (2000). Management of laparoscopic-related bowel injuries. Surg Endosc.

